# Realgar-induced apoptosis and differentiation in all-trans retinoic acid (ATRA)-sensitive NB_4_ and ATRA-resistant MR_2_ cells

**DOI:** 10.3892/ijo.2011.1276

**Published:** 2011-11-30

**Authors:** SIYU CHEN, YI FANG, LIHENG MA, SHANXI LIU, XINMIN LI

**Affiliations:** 1 Department of Oncology, Xin Hua Hospital Affiliated to Shanghai Jiao Tong University School of Medicine, Shanghai 200092; 2 State Key Laboratory for Medical Genomics, Shanghai Institute of Hematology, Rui Jin Hospital Affiliated to Shanghai Jiao Tong University School of Medicine, Shanghai 200025; 3 Department of Hematology, Ren Ji Hospital Affiliated to Shanghai Jiao Tong University School of Medicine, Shanghai 200127; 4 Department of Hematology, First Affiliated Hospital of Medical College of Xi'an Jiao Tong University, Xi'an 710061, P.R. China

**Keywords:** realgar, acute promyelocytic leukemia, apoptosis, differentiation, oligonucleotide microarray

## Abstract

Realgar has been used in Western medicine and Chinese traditional medicine since ancient times, and its promising anticancer activity has attracted much attention in recent years, especially for acute promyelocytic leukemia (APL). However, the therapeutic action of realgar treatment for APL remains to be fully elucidated. Cellular cytotoxicity, proliferation, apoptosis and differentiation were comprehensively investigated in realgar-treated cell lines derived from PML-RARα+ APL patient, including the all-trans retinoic acid (ATRA)-sensitive NB_4_ and ATRA-resistant MR_2_ cell lines. For analysis of key regulators of apoptosis and differentiation, gene expression profiles were performed in NB_4_ cells. Realgar was found to induce apoptosis and differentiation in both cell lines, and these effects were exerted simultaneously. Gene expression profiles indicated that genes influenced by realgar treatment were involved in the modulation of signal transduction, translation, transcription, metabolism and the immune response. Given its low toxicity, realgar is a promising alternative reagent for the therapy of APL. Our data contribute to an understanding of the underlying mechanism responsible for the therapeutic effects of realgar in the clinical treatment of APL.

## Introduction

In both Western and traditional Chinese medicine, arsenic has been used in antiseptics, antispasmodics and sedatives and for the treatment of ulcers and cancer since ancient times. There are mainly three types of mineral arsenicals; these include orpiment (As_2_S_3_), realgar (largely As_4_S_4_) and arsenolite (largely As_2_O_3_, arsenic trioxide, ATO). In the last decade, ATO has been proven to be effective in the treatment of malignant diseases, especially acute promyelocytic leukemia (APL). APL is a distinct subtype of acute myeloid leukemia (AML), and 98% of these patients have balanced reciprocal translocations between chromosomes 15 and 17 that result in the fusion of the promyelocytic leukemia (*PML*) and retinoic acid receptor α (*RARα*) gene.

Historically, APL was the most malignant form of acute leukemia due to the tendency for severe bleeding in patients. The complete remission (CR) rate following chemotherapy (CT) alone was 75% to 80% in newly diagnosed patients, and the 5-year disease-free survival (DFS) was as low as 35% (1–3). However, targeted therapy against the causative PML-RARα molecule has changed APL from a highly fatal disease to a highly curable one. All-trans retinoic acid (ATRA) was found to be the most effective differentiation therapy for APL, and this treatment raised the CR rate to between 90% and 95% and the 5-year DFS to 74% when combined with CT (4). ATO was found to improve the clinical outcome for newly diagnosed patients as well as for those who were relapsed, refractory to treatment or resistant to ATRA. When ATO was used as a single agent, the CR rate was 72.7% and 85.1% in the newly diagnosed and relapsed patients, respectively (5). When combined with ATRA in newly diagnosed APL patients, ATO increased the CR rate to 94.1% and the 5-year event-free survival (EFS) to 89.2% (6). Nevertheless, ATO is toxic and has severe side effects with long-term use, such as prolongation of the corrected QT interval and liver damage (7). Accordingly, ATO must be administered intravenously due to the potential for severe liver damage when given orally (7).

The efficacy of realgar has also been established for the treatment of APL and chronic myelogenous leukemia (CML) (8,9). Realgar is significantly less toxic than ATO and can be administered orally (10). Hence, realgar may provide an effective, safe and convenient way to treat malignant hematological diseases. The basis for the therapeutic effects of realgar, however, still needs to be fully elucidated. In this study, the effects of realgar on cellular cytotoxicity, proliferation, apoptosis and differentiation were comprehensively investigated using the PML-RARα-positive APL-derived ATRA-sensitive NB_4_ and ATRA-resistant MR_2_ cell lines. These data contribute to an understanding of the underlying mechanism responsible for the therapeutic effects of realgar in the clinical treatment of APL.

## Materials and methods

### Reagents

Highly purified realgar was prepared from mined natural realgar. The purity of As_4_S_4_ in our realgar preparation was not below 98.0%, which was confirmed by repeated X-ray powder diffraction analyses (in collaboration with the Research Center at the Xi'an Institute of Geology and Mineral Resources). These results were compatible with pure As_4_S_4_ standards and excluded the potential for trace amounts of ATO and other arsenic compounds that could influence the results. The high-purity realgar was dissolved in RPMI-1640 and sterilized by filtration. The content of As was determined by atomic absorption spectrometry. A 142.2 mg/l (near to 0.5 mM) stock solution was made by dilution in RPMI-1640 medium. According to the blood arsenic levels from As_4_S_4_-treated patients, the stock solution was appropriately diluted between 100 and 1000 times in RPMI-1640 for a working solution (8).

### Cell culture

The NB_4_ cell line was derived from a PML-RARα-positive APL patient, who was sensitive to ATRA. MR_2_, a subclone of NB_4_, is resistant to ATRA. Both cell lines were cultured in RPMI-1640 containing 10% fetal bovine serum (FCS; Gibco BRL, Gaithersburg, MD) in a humidified atmosphere with 5% CO_2_ at 37°C.

### LDH release assay

Following treatment with 355 μg/l realgar for 24 h, cell culture supernatants were collected for the LDH release assay (Cytotoxicity Detection kit; Roche, Indianapolis, IN, USA). Untreated NB_4_ cells that had been repeatedly freeze-thawed were set as the positive control for LDH release. Cells without any treatment or freeze-thaw cycle were used as the negative control.

### MTT assay

Briefly, cells were plated at a density of 2×10^4^ cells/100 μl/well in 96-well microtiter plates, and there were four replicates for each sample. Realgar (100 μl) at various concentrations was added to each well and was incubated for 24 h to 72 h. Cell culture medium without realgar was added to the control wells, and wells without cells were used as blank controls. Cells were then fed 20 μl/well MTT (5 mg/ml in PBS) and incubated for 4 h. After removing the supernatants, the MTT-formazan crystals were dissolved in 150 μl DMSO per well and the absorbance was measured at 570 nm using a multi-well plate reader (Model Anthos Labtec 2010.7 reader). The percent viability of each well was calculated as follows: The growth inhibition rate = (1-A_t_/A_c_) ×100%; (A_t_, average absorbance of test; A_c_, average absorbance of control).

### Cell morphology by light microscopy and transmission electron microscopy

For light microscopy, the cells were stained with Wright or hematoxylin-eosin (H&E) after they had been loaded onto slides by cytospin centrifugation (100 g, 4 min; Shandon, Runcorn, UK). One million cells were collected and prepared for transmission electron microscopy in the following order: cells were washed twice with phosphate-buffed saline (PBS, 0.01 M, pH 7.4), fixed in 2.5% glutaraldehyde (containing formamint) at 4°C for greater than 2 h, rinsed, post-fixed in 1% osmium tetroxide (OsO_4_) for 2 h, washed, dehydrated using a stepwise ethanol gradient and embedded in Epon 812 after permeation. The sections were stained with uranyl acetate followed by lead citrate and were observed using a transmission electron microscope.

### Annexin V staining

Following treatment with 355 μg/l of realgar for 36 h, 1×10^6^ cells were collected. The cells were washed in PBS and resuspended in 200 μl of staining solution containing 10 μl of annexin V-fluorescein isothiocyanate (FITC) and 5 μl of 20 μg/ml propidium iodide (PI). To distinguish live cells (negative for both fluorochromes) from apoptotic cells (positive for annexin V but negative for PI) and necrotic cells (positive for PI), 5,000 cells were analyzed by flow cytometry (Coulter EPICS Elite). All data were collected, stored and analyzed by Multigraph software (Coulter, Miami, FL).

### DNA content analysis

NB_4_ and MR_2_ cells were collected following treatment with 0, 177 μg/l, 355 μg/l or 711 μg/l of realgar for 24, 36, 48, 60 or 72 h. Cells were washed in PBS and fixed in 70% cold ethanol for at least 1 h at 4°C. Shortly before flow cytometry analysis, cells were rinsed with PBS, treated with 100 mg/l RNase A for at least 15 min at 37°C and stained with 20 mg/l PI. The distribution of cells having different DNA contents was analyzed by flow cytometry (Coulter EPICS Elite). Gating was performed to remove debris and doublets before collection. Data were measured using MultiCycle software (Phoenixm Flow Systems, San Diego, CA).

### NBT reduction assay

One million cells were harvested from control wells and wells treated with 0, 177 μg/l or 355 μg/l of realgar and 1 μM ATRA for 24, 48 or 72 h. NB_4_ cells treated with 1 μM ATRA were used as a positive control. Cells were washed and incubated in 1 ml PBS containing 1 mg/ml NBT and 5 μg/ml TPA for 30 min at 37°C. Cells containing intracellular black-blue formazan deposits were counted by microscopic examination and a minimum of 200 cells were examined in duplicate from four separate experiments. Following centrifugation, 600 ml DMSO was added to the cell pellets to solubilize the formazan deposits, and the amount of formazan was determined by recording the absorbance at 568.5 nm.

### CD11b and CD33 expression by flow cytometry

CD11b and CD33 expression was analyzed for NB_4_ and MR_2_ cells by flow cytometry. A volume of 100 μl of culture medium containing approximately 5×10^5^ cells was collected from control wells and from those treated with 177 μg/l or 355 μg/l of realgar and 1 μM ATRA for 48 h. NB_4_ cells treated with 1 μM ATRA were used as a positive control. Next, 15 μl of mouse anti-human CD11b-FITC or CD33-FITC antibody (Immunotech) was added to the cell medium and incubated for 30 min at room temperature. Mouse IgG1 isotype control-FITC (Immunotech) antibody was used as a negative control. Cells were analyzed by flow cytometry (Coulter EPICS Elite) following a wash with PBS and fixation in paraform.

### Immunofluorescence analysis of PML-RARα in NB_4_ cells

NB_4_ cells from untreated wells or from wells treated for 12 h with 1 μM realgar, ATO, As_2_S_2_, As_2_S_3_ and ATRA were centrifuged onto slides. The cells were fixed with methanol/acetone (1:1) at −20°C for 5 min, washed with PBS, blocked with BSA and incubated with mouse anti-human PML antibody (Santa Cruz, CA) and rabbit anti-mouse IgG-FITC (Dako, Denmark). Immunofluorescent images were recorded using a confocal microscope (Zeiss LSM 510, Germany).

### Oligonucleotide microarray analysis of NB_4_ cells

NB_4_ cells treated with 355 μg/l realgar for 4 h were compared to untreated controls. Then these two groups were further compared when pretreated with 10 mg/l cycloheximide for 1 h before any realgar treatment. The Biostar-1024D (HGEC-10d) microarrays consisted of 1003 novel or known genes (provided by United Gene Holdings Ltd., Shanghai). Construction of the microarray and the gene list used for this study followed the guidelines on the website http://www.unitedgene.com. RNA was isolated from NB4 cells. Purified mRNA was labelled with Cy3 or Cy5, then hybridized with microarray. The chip was scanned after rinsed. Each ratio of Cy5 to Cy3 was computed. Overall intensities were normalized for the correction coefficient of the natural logarithm ratio. To minimize artifacts arising from low-expression values, only genes with raw intensity values for both Cy3 and Cy5 >200 or those with >800 counts were chosen for differential analysis. A 2-fold difference in the ratio was used to represent differently expressed genes.

### Statistical analysis

All experiments were performed at least in triplicate, and the results were expressed as the mean ± SD. Statistical analyses were performed with the t-test, χ^2^ test and the multiple comparisons test using SPSS12.0 software.

## Results

### Cytotoxicity assay

An increase in LDH release indicates a breakdown of the cell membrane that can lead to cell death. In this study, we compared the LDH activity of NB_4_ cells treated for 24 h with 355 μg/l realgar with that of the untreated controls. However, there was no significant difference in activity between these two groups (67±3.5 U/l vs. 69±2.7 U/l, P>0.05). The activity from the positive control was 282±7.2 U/l, while that from the negative control was 48 U/l. These results suggest that there was no increase in membrane permeability in NB_4_ cells following treatment with 355 μg/l realgar.

### Cell proliferation

**Figure 1:**
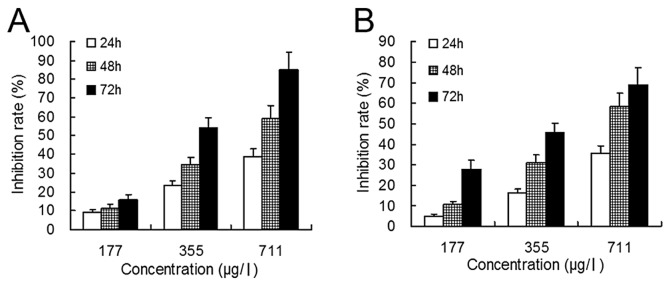
The inhibition rates following realgar treatment in NB_4_ and MR_2_ cells from the MTT assay. (A) NB_4_ and (B) MR_2_ were treated with three different concentrations of realgar: 177 μg/l, 355 μg/l and 711 μg/l.

Cell proliferation was evaluated by the MTT assay. When NB_4_ and MR_2_ cells were treated with realgar at concentrations of 177 μg/l, 355 μg/l and 711 μg/l for 24 h, the growth inhibition rates were 9.4±1.0%, 23.4±2.4% and 38.9±4.1% for NB_4_ cells (P<0.01) and were 4.9±1.0%, 16.2±1.8% and 35.5±3.7% for MR_2_ cells (P<0.01), respectively (Fig. 1). However, following treatment with 44–88 μg/l realgar, the inhibition rates of these two groups of cells were negligible (data not shown).

### Cell apoptosis and differentiation

#### Cell morphology

**Figure 2:**
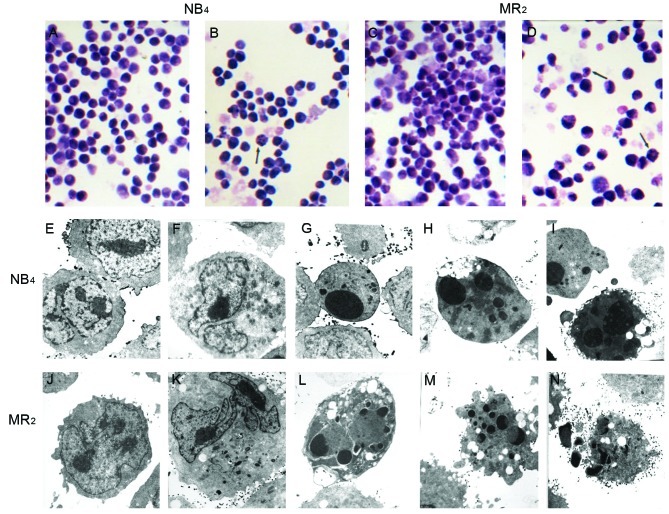
Cell morphology of NB_4_ and MR_2_ under microscopy. (A) Untreated NB_4_ and (C) MR_2_ cells with H&E stain by light microscopy. (B) Treated NB_4_ and (D) MR_2_ cells with H&E stain by light microscopy treated with 177 μg/l realgar for 60 h. (E) Untreated NB_4_ and (J) MR_2_ cells by transmission electron microscopy. (F-I) Treated NB_4_ and (K-N) MR_2_ cells by transmission electron microscopy treated with 355 μg/l realgar for 48 and 65 h, respectively.

By light microscopy, NB_4_ and MR_2_ cells showed reduced cell size, condensed nuclei and dark staining, which are indicative of apoptotic changes to cell morphology. The condensed nuclei broke into scattered small lumps surrounding the cell membrane. Following treatment with 177 μg/l of realgar, both cell populations began to differentiate towards having larger areas of clear cytoplasm and hollow nuclei. This effect was more obvious in 60 h for both cell lines (Fig. 2B and D). By transmission electron microscopy, apoptotic changes in NB_4_ (Fig. 2G-I) and MR_2_ (Fig. 2L-N) cells were obvious after exposure to 355 μg/l realgar for 48 h and 65 h, respectively. These changes were characterized by the presence of apoptotic bodies, cell shrinkage, vacuole formation and undulations of the plasma membrane. Additionally, a certain extent of differentiation was suggested by the decreased ratio of nuclei to plasma, by the emergence of some cellular particles, by the partial disappearance of nucleoli and by nuclear remodeling, which ranged from simple indentations to polylobular nuclei (Fig. 2F and K).

#### Annexin V staining

Annexin V is expressed on the membranes of apoptotic cells in the early stages of apoptosis. After treatment with 355 μg/l of realgar for 36 h, the percents of NB_4_ and MR_2_ cells expressing Annexin V (PI^−^) were 10.4±0.5% and 16.2±0.6%, while those of the blank controls were 0.5±0.1% and 0.3±0.1%, respectively (both P<0.005).

#### DNA content analysis

**Figure 3:**
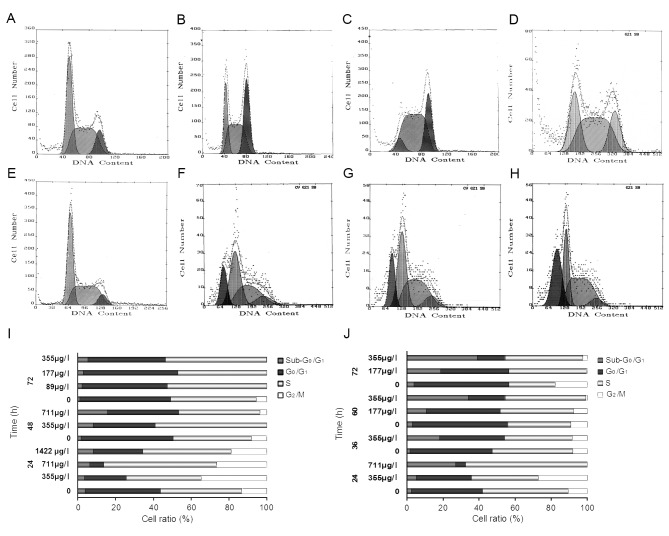
The results of DNA content analyses following realgar treatment. NB_4_ cells (I): untreated (A), 355 μg/l realgar (B), 711 μg/l realgar (C) and 1422 μg/l realgar (D) for 24 h, MR2 cells (J): untreated (E), 177 μg/l realgar for 60 h (F), 355 μg/l realgar for 36 h (G), 355 μg/l realgar for 60 h (H).

After NB_4_ cells were treated for 24 h, we found evidence for significant cell cycle arrest at the G_2_/M stage with 355 μg/l or 711 μg/l realgar. Compared with the untreated group, this change in the cell cycle distribution was significant (P<0.005). But it did not increase in significance for the treatment of 1422 μg/l realgar (Fig. 3A-D). In MR_2_ cells treated with 177 μg/l or 355 μg/l realgar for 36 h or 60 h, the fraction of cells in the G_0_/G_1_ stage was sharply reduced, while that in S phase was remarkably increased (P<0.005) (Fig. 3E-H). Additionally, in both cell lines, the sub-G_0_/G_1_ cells increased in a time- and dose-dependent manner (Fig. 3I and J).

#### NBT reduction assay

**Figure 4:**
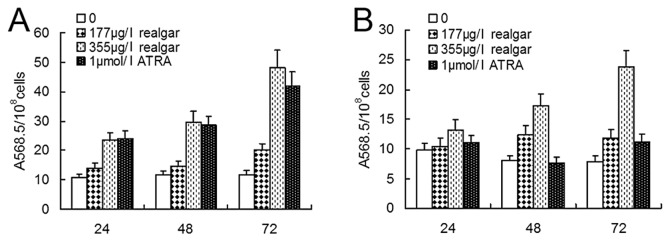
The results of NBT reduction assays. (A) Absorbance rates at the wavelength of 568.5 nm in NB_4_ and (B) MR_2_ cell lines untreated or treated with 177 μg/l, 355 μg/l realgar and 1 μM ATRA for 24 h, 48 h and 72 h, respectively.

Realgar-induced differentiation was assessed by the NBT reduction assay. Among NB_4_ cells, NBT-positive cells were greatly increased following treatment with 1 μM ATRA, 177 μg/l or 355 μg/l realgar for 24, 48 and 72 h (all P<0.005) (Fig. 4A). Compared with 1 μM ATRA treatment, however, the increase was not significant for the treatment with 177 μg/l realgar at these time points (P<0.005). For cells treated with 355 μg/l realgar, there was no difference at 24 h or 48 h (P>0.05) in comparison to those given ATRA treatment, but the percentage of NBT-positive cells was significantly greater at 72 h (P<0.0005). For MR_2_ cells, no changes occurred following 1 μM ATRA treatment for 24, 48 or 72 h (P>0.05). In samples treated with 177 μg/l realgar, NBT-positive cells were not increased at 24 h (P>0.05) but were increased at 48 h (P<0.01) and 72 h (P<0.05). However, treatment of cells with 355 μg/l realgar increased the NBT positive percentages at 24 h (P<0.05), 48 h (P<0.001) and 72 h (P<0.0005) (Fig. 4B).

#### CD11b and CD33 expression

**Figure 5:**
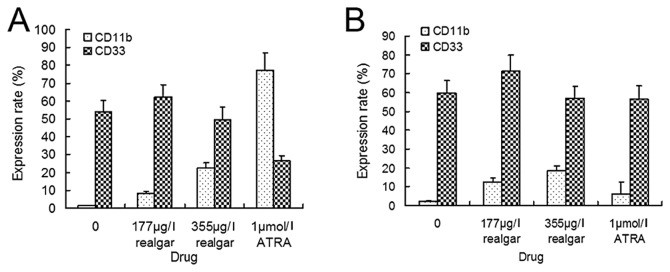
The result of CD11b and CD33 expression. The expression rate of CD11b and CD33 in (A) NB_4_ and (B) MR_2_.

CD11b is expressed later in monocytic or granulocytic differentiation, whereas CD33 is associated with primitive myeloid cell populations in APL. FACS analysis of NB_4_ cells, following treatment with 1 μM ATRA, found the expression of CD11b to be significantly increased (P<0.005) but that of CD33 to be decreased (P<0.01). Treatment with 177 μg/l or 355 μg/l realgar was also found to increase the expression of CD11b (P<0.05 and P<0.005, respectively). However, treatment with either of these concentrations of realgar resulted in weaker expression than did treatment with ATRA (P<0.005). Unlike ATRA, realgar treatment did not cause a significant decrease in the proportion of cells that expressed CD33 (P>0.05) (Fig. 5A). In the ATRA-resistant MR_2_ cells, 1 μM ATRA neither increased the expression of CD11b significantly (P>0.05) nor had an effect on the expression of CD33 (P>0.05). Following treatment with 177 μg/l and 355 μg/l realgar, however, the expression of CD11b was significantly increased (P<0.005). Additionally, realgar treatment did not decrease the expression of CD33 (P>0.05) (Fig. 5B).

#### Localization of PML-RARα protein in realgar-treated NB_4_ cells

**Figure 6:**
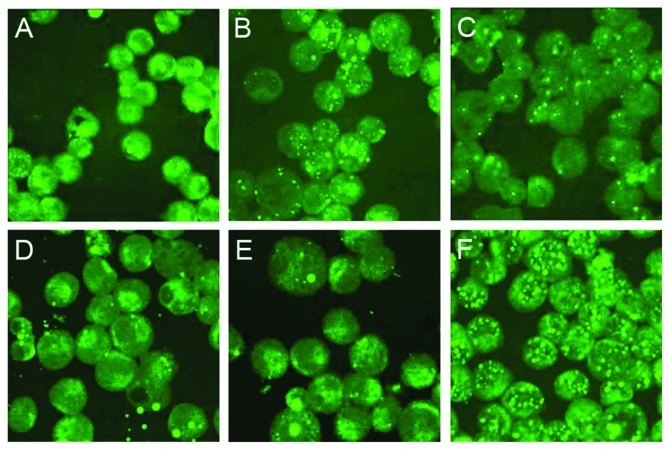
Immunofluorescence analysis of PML-RARα in NB_4_. (A) Untreated NB_4_ cells as blank control; (B) 1 μM As_2_S_2_; (C) 1 μM (300 μg/l) realgar; (D) 1 μM As_2_O_3_; (E) 1 μM As_2_S_3_ and (F) 1 μM ATRA.

Using confocal microscopy, the localization of PML-RARα protein was found to be altered in realgar-treated NB_4_ cells. In these cells, most of the PML-RARα protein had entered the nuclei and PML-positive particles had accumulated. Similar relocalization was also observed in cells treated with ATO, As_2_S_2_, As_2_S_3_ and ATRA (Fig. 6).

#### Gene expression profile in NB_4_ cells

We performed microarray analyses to evaluate the global gene expression profiles of NB_4_ cells treated with 355 μg/l realgar for 4 h. Compared with the untreated controls, gene expression profiling revealed 13 aberrantly expressed genes in treated cells. Of these, the following 11 genes were down-regulated: the signal transduction genes U51903 (*IQGAP2*), L42572 (*IMMT*) and Y00483 (*GPX1*); the protein translation genes M86752 (*STIP1*) and U58048 (*CHMP1A*); the metabolism genes X66435 (*HMGCS1*) and D16480 (*HADHA*); the DNA binding and transcription factor gene AF036613 (*GTF2IP1*); the immune-related gene NM_006995 (*BTN2A2*); and two unclassified genes, AF052108 (*LOC157627*) and D21261 (*TAGLN2*) (Table I). In comparison, Z22533 (*ACVRL1*), a signal transduction gene, and NM_007057 (*ZWINT*), were up-regulated in treated cells. When these two groups were compared after pretreatment with 10 mg/l cycloheximide for 1 h, we found that the genes U51903 (*IQGAP2*), X66435 (*HMGCS1*) and AF036613 (*GTF2IP1*) were down-regulated, whereas the gene Z22533 (*ACVRL1*) was up-regulated. These changes in gene expression were independent of the synthesis of new protein.

## Discussion

Realgar has been used in Chinese traditional medicine for more than 1500 years. In recent years, its promising anti-cancer potential has attracted attention at home and abroad, especially in the therapy of APL. According to a pilot report on As_4_S_4_ levels in APL by Lu *et al* (8), realgar used alone as therapy was effective in inducing hematological CR (HCR) in both newly diagnosed and relapsed APL patients. Additionally, cytogenetic and molecular CR was obtained for 87.5% of the newly diagnosed patients and for 5 of the 7 patients with hematologic relapses. Furthermore, realgar treatment is highly effective for CR maintenance, as there was a 3-year DFS of 76.6% for the newly diagnosed patients and a 6-year DFS of 87.4% for the HCR patients.

To fully elucidate the therapeutic basis of realgar in APL, PML-RARα+ cell line ATRA-sensitive NB_4_ and ATRA-resistant MR_2_ were investigated in this study. After being treated with realgar in MTT assay, both cells were significantly inhibited in a dose- and time-dependent manner. An LDH-releasing assay demonstrated that realgar had no direct cytotoxic effect on the cell membrane, which was consistent with the clinical observation that no obvious myelosuppression was observed in APL patients treated with realgar (8).

The mechanism of action of realgar was also studied in NB_4_ and MR_2_ cells. ATO treatment was found to exert dual effects on APL cells in a dose-dependent manner *in vitro*. At greater concentrations (0.5–2.0 μM), ATO has been shown to trigger apoptosis, while at lower concentrations (0.1–0.5 μM), it can induce partial differentiation (11). The dual effects of ATO were reported in the bone marrow of APL patients and in the PML-RARα/APL mouse model (12). In this study, the dual effects of realgar were demonstrated in ATRA-sensitive NB_4_ and ATRA-resistant MR_2_ cells. In contrast to ATO treatment, the dual effects of realgar treatment were exerted simultaneously. Morphologically, we found that with greater realgar concentration or culture time, more apoptotic cells were observed. Simultaneously, partial differentiation was indicated by the decreased nuclear to cytoplasm ratio, the partial disappearance of nucleoli and the nuclear remodeling ranging from indentation to polylobular nuclei. Apoptotic effects of treatment were demonstated by the significant increase in the percentage of cells expressing Annexin V (PI^−^) and the sub-G_0_/G_1_ cells increased in a time- and dose-dependent manner in DNA content assay. As evidence of cellular differentiation, the NBT reduction values were enhanced in NB_4_ and MR_2_ cells treated with realgar. Realgar treatment did not reduce the expression of CD33. However, it did significantly induce the expression of CD11b in NB_4_ and MR_2_ cells, which suggested the partial differentiation of these two APL cell lines. Compared to ATRA treatment, realgar treatment exerted a weaker differentiation effect on NB_4_ cells, while ATRA did not have any differentiation effect on MR_2_ cells. Similar to ATO treatment, realgar treatment effectively changed the localization of PML-RARα protein to the nuclei and increased PML positive particles in NB_4_ cells (13). This relocalization was also detected in the bone marrow cells of APL patients treated with arsenic sulfides, although this occurred before any morphologic change was detected (8).

Cai *et al* reported that 0.1–0.5 mM ATO did not induce differentiation in ATRA-resistant cell lines (14). In our study, we found that realgar induced differentiation in MR_2_ cells. This result indicated that some different mechanisms may underlie between the differentiation effect of ATO and realgar. Based on the simultaneous dual effect of realgar, we hypothesized that apoptosis and differentiation might share some initiating steps or signal transduction in APL cells.

To further define the molecular mechanisms at work following realgar treatment, microarrays from NB_4_ cells found 11 genes down-regulated and two genes up-regulated. The activity of 4 of these 13 genes was not blocked by the addition of cycloheximide, which suggests that these 4 genes were of importance because their modulation was independent of new protein synthesis. These 13 genes are known to be involved in the modulation of signal transduction, translation, transcription and metabolism and the immune response. Among them, U51903 encodes the Ras GTPase-activating-related human protein IQGAP2, which inhibits both the intrinsic and the RhoGAP-stimulated GTP hydrolysis rates of Cdc42 and Rac1 by binding Cdc42 and Rac1, but not RhoA (15). The down-regulation of U51903 by realgar led to the decreased expression of IQGAP2, which may up-regulate Cdc42 and Rac1 to activate the JNK pathway leading to apoptosis. In ATO-induced apoptosis, JNK signaling is also activated (16). The gene Z22533 encodes the activin receptor-like kinase ALK1, a member of serine/threonine protein family, which is in the transforming growth factor-β (TGF-β) receptor superfamily (17). Realgar treatment up-regulated Z22533 and the expression of ALK1, which may inhibit cell proliferation by regulating TGF-β/Smad signaling pathway. Recently, a proteomic investigation of cellular differentiation caused by As_4_S_4_ was performed in the retinoic acid (RA)-resistant cell line NB_4_-R1 using high-solution two-dimensional electrophoresis and mass spectrometry. These results suggested that the proteins SET, RPP2 and PHB may be novel effective therapeutic targets for RA-resistant APL (18).

In patients, realgar treatment was well tolerated and had only moderate side effects, such as transient liver injury and elongated QT intervals without symptoms (8). No obvious myelosuppression or serious cardiac events were observed in APL patients treated with realgar. However, treatment with ATRA and ATO was found to induce an ATRA syndrome that was severe and was responsible for deaths early during remission induction (8). Recently, realgar nanoparticles have been studied to enhance bioavailability (19,20). Accordingly, realgar may provide an effective, safe and convenient way to treat APL patients.

In conclusion, in the present study, realgar was found to mediate both apoptotic and differentiation effects in the ATRA-sensitive NB_4_ and ATRA-resistant MR_2_ PML-RARα + APL cell lines. Dual effects were exerted simultaneously and included the modulation of signal transduction, translation, transcription, metabolism and immune response genes. Given its low toxicity, realgar is a promising alternative reagent for the therapy of APL. Our data contribute to the understanding of the underlying mechanisms of realgar treatment and its clinical application for APL.

## Figures and Tables

**Table I:** Differentially expressed genes in NB_4_ cells following treatment with realgar.

				Ratio (Cy5/Cy3^a^)			
Gene ID	GenBank ID	Definition	Category	Chip1		Chip2	
10788	U51903^b^	IQGAP2, IQ motif containing GTPase activating protein 2 [*Homo sapiens*]	Signal transduction	0.236	0.276	0.261	0.268
2970	AF036613^b^	GTF2IP1, general transcription factor IIi pseudogene 1 [*Homo sapiens*]	DNA transcription, TF	0.276	0.265	0.461	0.453
10989	L42572	IMMT, inner membrane protein, mitochondrial [*Homo sapiens*]	Signal transduction/cytoskeletons	0.324	0.333	0.492	0.513
3157	X66435^b^	HMGCS1, 3-hydroxy-3-methylglutaryl-CoA synthase 1 (soluble) [*Homo sapiens*]	Metabolism	0.351	0.320	0.227	0.248
8407	D21261	TAGLN2, transgelin 2 [*Homo sapiens*]		0.367	0.395	0.637	0.652
10963	M86752	STIP1, stress-induced-phosphoprotein 1 [*Homo sapiens*]	Translation, synthesis of protein	0.380	0.411	0.561	0.678
2876	Y00483	GPX1, glutathione peroxidase 1 [*Homo sapiens*]	Signal transduction	0.409	0.427	0.779	0.886
157627	AF052108	LOC157627, uncharacterized LOC157627 [*Homo sapiens*]		0.403	0.456	0.553	0.531
3030	D16480	HADHA, hydroxyacyl-CoA dehydrogenase/3-ketoacyl-CoA thiolase/enoyl-CoA hydratase (trifunctional protein), α-subunit [*Homo sapiens*]	Metabolism	0.422	0.439	0.622	0.686
5119	U58048	CHMP1A, charged multivesicular body protein 1A [*Homo sapiens*]	Transportation of proteins	0.466	0.500	0.785	0.800
10385	NM_006995	BTN2A2 butyrophilin, subfamily 2, member A2 [*Homo sapiens*]	Immune response	0.487	0.481	0.657	0.631
11130	NM_007057	ZWINT, ZW10 interacter [*Homo sapiens*]		2.103	2.008	0.958	0.995
94	Z22533^b^	ACVRL1, activin A receptor type II-like 1 [*Homo sapiens*]	Signal transduction	2.262	2.170	2.749	2.672

a Normalized Cy3;

b The transcriptional regulation of 4 out of the 13 genes could not be blocked by the pre-treatment of cycloheximide.

## References

[1] Cunningham I, Gee TS, Reich LM, Kempin SJ, Naval AN, Clarkson BD (1989). Acute promyelocytic leukemia: treatment results during a decade at Memorial Hospital. Blood.

[2] Ribeiro RC, Rego E (2006). Management of APL in developing countries: epidemiology, challenges and opportunities for international collaboration. Hematology Am Soc Hematol Educ Program.

[3] Sanz MA, Jarque I, Martin G (1988). Acute promyelocytic leukemia. Therapy results and prognostic factors. Cancer.

[4] Wang ZY, Chen Z (2008). Acute promyelocytic leukemia: from highly fatal to highly curable. Blood.

[5] Niu C, Yan H, Yu T (1999). Studies on treatment of acute promyelocytic leukemia with arsenic trioxide: remission induction, follow-up, and molecular monitoring in 11 newly diagnosed and 47 relapsed acute promyelocytic leukemia patients. Blood.

[6] Hu J, Liu YF, Wu CF (2009). Long-term efficacy and safety of all-trans retinoic acid/arsenic trioxide-based therapy in newly diagnosed acute promyelocytic leukemia. Proc Natl Acad Sci USA.

[7] Liu J, Lu Y, Wu Q, Goyer RA, Waalkes MP (2008). Mineral arsenicals in traditional medicines: orpiment, realgar, and arsenolite. J Pharmacol Exp Ther.

[8] Lu DP, Qiu JY, Jiang B (2002). Tetra-arsenic tetra-sulfide for the treatment of acute promyelocytic leukemia: a pilot report. Blood.

[9] Mao JH, Sun XY, Liu JX (2010). As4S4 targets RING-type E3 ligase c-CBL to induce degradation of BCR-ABL in chronic myelogenous leukemia. Proc Natl Acad Sci USA.

[10] Liu J, Liang SX, Lu YF, Miao JW, Wu Q, Shi JS (2011). Realgar and realgar-containing Liu-Shen-Wan are less acutely toxic than arsenite and arsenate. J Ethnopharmacol.

[11] Chen GQ, Shi XG, Tang W (1997). Use of arsenic trioxide (As2O3) in the treatment of acute promyelocytic leukemia (APL): I. As2O3 exerts dose-dependent dual effects on APL cells. Blood.

[12] Chen Z, Zhao WL, Shen ZX (2007). Arsenic trioxide and acute promyelocytic leukemia: clinical and biological. Curr Top Microbiol Immunol.

[13] Chen GQ, Zhu J, Shi XG (1996). In vitro studies on cellular and molecular mechanisms of arsenic trioxide (As2O3) in the treatment of acute promyelocytic leukemia: As2O3 induces NB4 cell apoptosis with downregulation of Bcl-2 expression and modulation of PML-RAR alpha/PML proteins. Blood.

[14] Cai X, Shen YL, Zhu Q (2000). Arsenic trioxide-induced apoptosis and differentiation are associated respectively with mitochondrial transmembrane potential collapse and retinoic acid signaling pathways in acute promyelocytic leukemia. Leukemia.

[15] Brill S, Li S, Lyman CW (1996). The Ras GTPase-activating-protein-related human protein IQGAP2 harbors a potential actin binding domain and interacts with calmodulin and Rho family GTPases. Mol Cell Biol.

[16] Davison K, Mann KK, Miller WH (2002). Arsenic trioxide: mechanisms of action. Semin Hematol.

[17] Ten Dijke P, Ichijo H, Franzen P (1993). Activin receptor-like kinases: a novel subclass of cell-surface receptors with predicted serine/threonine kinase activity. Oncogene.

[18] Qi J, He P, Chen W, Wang H, Wang X, Zhang M (2010). Comparative proteome study of apoptosis induced by As4S4 in retinoid acid resistant human acute promyelocytic leukemia NB4-R1 cells. Leuk Res.

[19] Wu JZ, Ho PC (2006). Evaluation of the in vitro activity and in vivo bioavailability of realgar nanoparticles prepared by cryo-grinding. Eur J Pharm Sci.

[20] Xi RG, Huang J, Li D, Wang XB, Wu LJ (2008). Roles of PI3-K/Akt pathways in nanoparticle realgar powders-induced apoptosis in U937 cells. Acta Pharmacol Sin.

